# Risk factors for poor outcomes in patients with acute lower leg compartment syndrome: a retrospective study of 103 cases

**DOI:** 10.1186/s13018-024-04719-7

**Published:** 2024-04-20

**Authors:** Zhe Lin, Zhiyong Hou, Jialiang Guo, Yongsheng Lin, Yingze Zhang

**Affiliations:** 1 Department of Orthopaedic Surgery, The Third Hospital of Hebei Medical University, Shijiazhuang, China; 2 Key Laboratory of Orthopaedic Biomechanics of Hebei Province, Shijiazhuang, China; 3 Orthopaedic Research Institution of Hebei Province, Hebei, China; 4 Chinese Academy of Engineering, Beijing, China; 5 The Third Affiliated Hospital, Hebei Medical University, Shijiazhuang, 050051 Hebei China

**Keywords:** Acute compartment syndrome, Crus, Fasciotomy, Arterial injury

## Abstract

**Purpose:**

The primary aim of this study was to investigate the risk factors associated with poor outcomes following acute compartment syndrome (ACS) of lower leg. The secondary objective was to determine if delayed fasciotomy is linked to poor outcomes.

**Methods:**

In this retrospective case control study approved by the institutional review board, we identified 103 patients with ACS of the lower leg. Poor outcome was defined as a composite variable that included limb amputation, neurological deficit and contracture. Among these, 44 patients exhibited poor outcome while 59 patients demonstrated a good outcome. Patient-related factors, laboratory values, and treatment-related factors were analyzed using electronic medical records. Univariate statistical and logistic regression analyses were conducted to determine significance.

**Results:**

Bivariate analyses showed that the mechanism of injury (*P =* 0.021), open injury (*P =* 0.001), arterial injury (*P*<0.001), hemoglobin levels (HB) (*P <* 0.001), white blood cell count (WBC) (*P* = 0.008), albumin levels (ALB) (*P*<0.001), creatine kinase levels (CK) at presentation (*P =* 0.015), CK at peak (*P*<0.001), creatine kinase levels (Ca) (*P* = 0.004), dehydrating agent (*P* = 0.036), and debridement (*P* = 0.005) were found to be associated with the risk of poor outcomes. Logistic regression analyses revealed that arterial injury [ *P*< 0.001, OR = 66.172, 95% CI (10.536, 415.611)] was an independent risk factor for poor outcomes. However, HB [*P* = 0.005, OR = 0.934, 95% CI (0.891, 0.979)] was a protective factor against poor outcomes. Receiver operating characteristic (ROC) curve analysis showed that the cut-off values of HB to prevent poor outcome following ACS was 102.45 g/L.

**Conclusions:**

ACS of the lower leg is a serious complication often associated with a poor prognosis. Patients with arterial injury or lower HB have a significantly increased risk of having poor outcomes. Poor outcomes were not found to be associated with the timing of fasciotomy in this study.

## Introduction

Acute compartment syndrome (ACS) is an orthopedic emergency that most frequently originates from trauma [1–6]. It can occur in the upper limb or the lower limb, although it most commonly affects the lower leg [2–10]. ACS is characterized by increased intercompartmental pressure and decreased tissue perfusion following progressive tissue edema, internal bleeding, or overly tight plaster fixation [2, 3, 6, 11–13]. Since persistent limb ischemia caused by ACS can lead to irreversible muscle necrosis and nerve damage, missed diagnosis and delayed treatment have serious and sometimes catastrophic consequences for patients [14]. During the acute period, a non-salvageable limb may cause life-threatening conditions, such as kidney failure, hyperkalemia, metabolic acidosis or septic shock, leading the treating surgeon to perform primary limb amputation [4, 7, 15]. Sequelae following limb salvage, for example, Volkmann contracture and neurologic dysfunction, can also severely impede limb function [7, 8, 16].

Early fasciotomy is generally considered to be the most critical treatment for preventing poor outcomes [5, 6, 16, 17]. A fascial incision can release the intra-compartmental pressure and restore tissue perfusion. However, fasciotomy is associated with complications, including delayed wound closure, cosmetic problems, delayed union or nonunion of fractures, pain, nerve injury, permanent muscle weakness, and chronic venous insufficiency [2, 10, 18–20]. However, in recent years, some studies have reported that time to fasciotomy was not associated with poor outcomes in patients with ACS [7, 17, 21].

The primary objective of this study was to investigate the risk factors for poor outcomes in patients with ACS in the lower leg. The early identification and timely treatment of risk factors strongly influence the development of ACS in the lower leg. The secondary objective of this study was to determine whether delayed fasciotomy is associated with poor outcomes in patients with ACS of the lower leg.

## Methods

This retrospective case control study received approval from the Institutional Review Board of our hospital (NCT04529330, S2020-022–1) prior to data collection. The study was conducted according to STROBE guidelines.Medical records from our Level I trauma center from May 2007 to July 2023 were retrospectively reviewed. We identified patients with acute compartment syndrome of the lower leg by querying our hospital’s medical records database using International Classification of Disease, 9th Revision (ICD-9) codes 729.72 (nontraumatic compartment syndrome of lower extremity) and 958.92 (traumatic compartment syndrome of lower extremity). Since the ICD-9 diagnosis codes pertain to the lower limb and not specifically to the lower leg, patients with lower limb diagnoses other than acute lower leg compartment syndrome, including gluteal compartment syndrome, thigh compartment syndrome, foot compartment syndrome, and chronic exertional compartment syndrome, were excluded. To avoid bias, we established the following criteria.

### Inclusion criteria

1. Clear diagnosis of ACS of lower extremity.
2. Age greater than 18 years.

### Exclusion criteria

3. Patients suffered ACS in lower extremity, excluding lower leg.
4. Patient without adequate medical records.
5. Patient death during hospitalization or after discharge.
6. Patient refusal of surgery or other treatment.
7. Patient initially treated with fasciotomies at an outside hospital.
8. More than 72 h elapsed from the injury to arrival at the hospital.
9. Chronic exertional compartment syndrome.

A poor outcome was defined as a composite variable that included one or more of the following: (1) limb amputation, (2) neurological deficit, or (3) contracture. Patients with poor outcomes were classified as belonging to the poor outcome group (PG), while those without poor outcomes were classified as belonging to the good outcome group (GG). Patient-related factors, laboratory values and treatment-related factors were collected in this study.

Patient-Related Factors:

The following patient-related factors were examined: age, sex, smoking status (yes vs. no), mechanism of injury, open injury (yes or no), presence of fracture (yes vs. no), presence of a blister (yes vs. no), and occurrence of arterial injury (yes vs. no). The mechanisms of injury were further classified as low-energy blunt trauma, high-energy blunt trauma, crush injury, or others (including penetrating trauma, carbon monoxide poisoning, and arterial thrombosis). The arterial injuries observed included those of the iliac artery, lateral iliac artery, femoral artery, popliteal artery, anterior tibial artery, and posterior tibial artery.

### Laboratory Values

The following laboratory values were assessed: hemoglobin levels (HB, g/L), platelet count (PLT, 10^9^/L), international normalized ratio (INR, s), white blood cell count (WBC, 10^9^/L), prothrombin time (PT, s), activated partial thromboplastin time (APTT, s), fibrinogen level (FIB, g/L), alanine aminotransferase level (ALT, U/L), aspartate aminotransferase level (AST, U/L), albumin level (ALB, g/L), glucose level (GLU, mmol/L), creatine kinase levels (CK, U/L) at presentation and peak, sodium level (Na, mmol/L), potassium level (K, mmol/L), and calcium level (Ca, mmol/L). Except for CK at peak, all other laboratory values were collected for the first time after the patient’s visit.

### Treatment-related factors

The following treatment-related factors were examined: application of a dehydrating agent (yes vs. no), use of negative pressure wound vacuum-assisted closure (VAC, yes vs. no), number of debridement procedures performed on the affected limb (0, 1 ∼ 2, ≥ 3), hospital day (HOD, day), time from injury to hospital admission (hours), and time from injury to fasciotomy (less than 6 h, 6–24 h, or greater than 24 h). Previous studies have identified 6 h and 24 h as important benchmarks in acute compartment syndrome [7, 16, 21, 22].

All patient data were organized and analyzed using SPSS (version 25.0 SPSS, Inc., Chicago, IL). For measurement data, if the data had a normal distribution, the data are presented as the mean ± SD (standard deviation) and were analyzed with t tests; if not, the data are presented as the median (interquartile range), and the Mann–Whitney U test was used to perform statistical comparisons between groups. Comparisons of categorical data were performed using the chi-square test. Logistic regression analysis was also conducted to assess patient outcomes. The significance level was set at α = 0.05, and a *p* value less than 0.05 was used to indicate statistical significance. Receiver operating characteristic (ROC) curve analysis was conducted to show the cut-off value, sensitivity and specificity of measurement data.

## Results

**Fig. 1:**
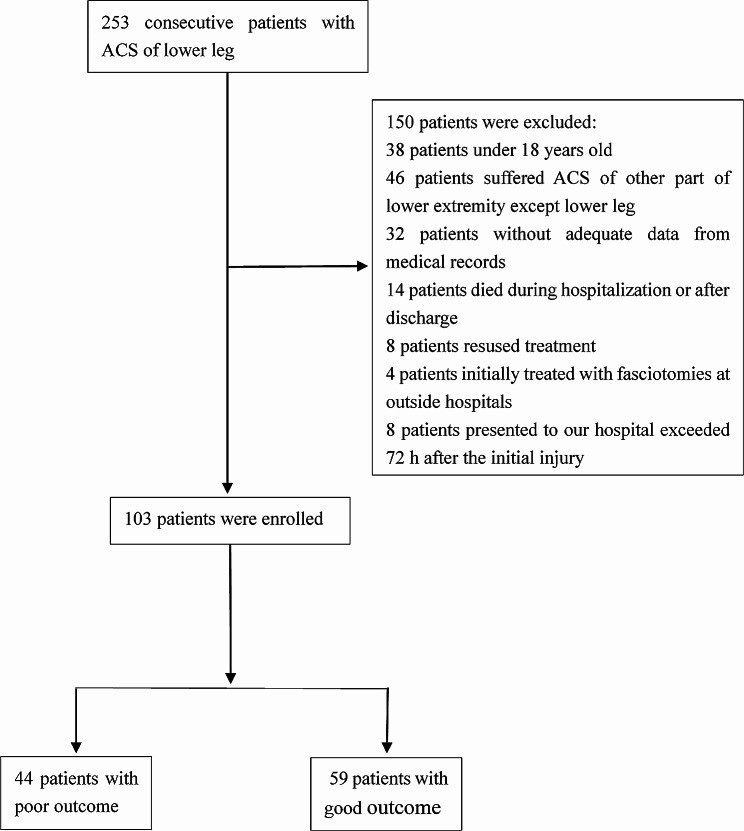
Flow diagram of included patients

**Fig. 2:**
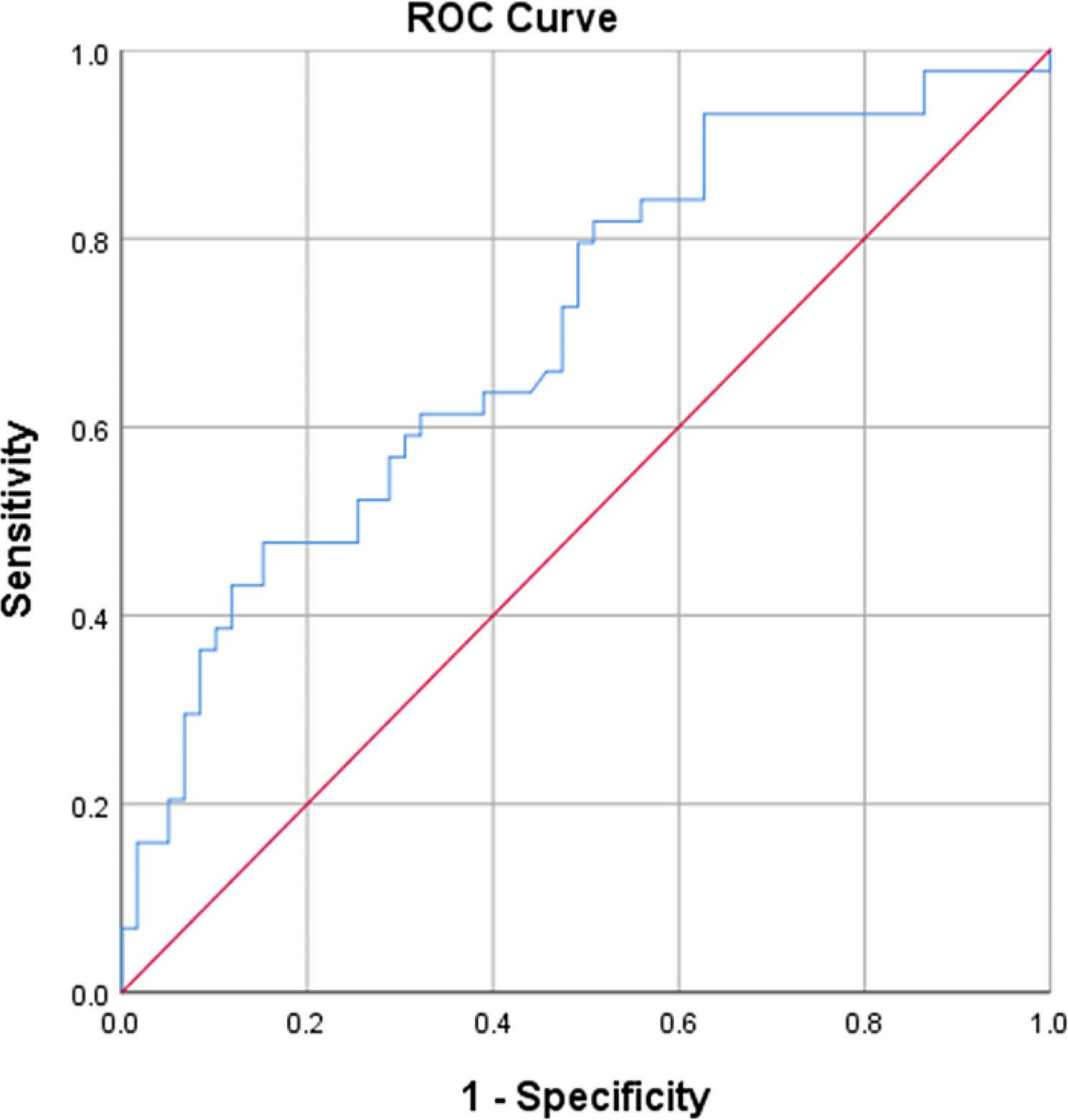
ROC curve of HB

The initial query yielded 253 patients with ACS in the lower leg. There were 150 patients who were excluded from our study protocol according to the exclusion criteria. Among these patients, 38 patients were younger than 18 years, 46 patients suffered ACS in lower extremity, excluding lower leg, 32 patients did not have adequate data from the medical records, 14 patients died during hospitalization or after discharge, 8 patients refused treatment, 4 patients were initially treated with fasciotomies at outside hospitals, and 8 patients presented to our hospital more than 72 h after the initial injury. Ultimately, 103 patients were included in the study after applying the exclusion criteria (Fig. 1). 94 patients were men, and 9 were women. The average age was 41 years (range 18–75 years). 4 patients developed a double-sided syndrome in which fasciotomies were performed on both legs, for a total of 107 lower legs with ACS. Poor outcomes occurred in 44 (43.7%) of the 103 patients. Bivariate analyses showed that the mechanism of injury (*P =* 0.021), open injury (*P =* 0.001), arterial injury (*P*<0.001), HB (*P <* 0.001), WBC (*P =* 0.008), ALB (*P*<0.001), CK at presentation (*P =* 0.015), CK at peak (*P*<0.001), Ca (*P =* 0.004), dehydrating agent (*P =* 0.036), and debridement (*P =* 0.005) were found to be associated with the risk of poor outcomes (Tables 1 and 2). Logistic regression analyses revealed that arterial injury [ *P*< 0.001, OR = 66.172, 95% CI (10.536, 415.611)] was an independent risk factor for poor outcomes. However, HB [*P* = 0.005, OR = 0.934, 95% CI (0.891, 0.979)] was a protective factor against poor outcomes (Table 3). Receiver operating characteristic (ROC) curve analysis showed that the cut-off values of HB to prevent poor outcome following ACS was 102.45 g/L (Table 4; Fig. 2).

Regarding the time to fasciotomy, 9.7% of patients (10 out of 103) underwent emergency fasciotomy within 6 h after injury, 61.2% (63 out of 103) underwent the procedure between 6 and 24 h after injury, and 29.1% (30 out of 103) underwent it more than 24 h after injury. We did not find an association between the time to fasciotomy (*P* = 0.252) and poor outcomes in the bivariate analyses.

**Table 1:** Bivariate Analyses of Count Data in Patients with ACS of lower leg

Item	Type	Good prognosis group (*n* = 59)	Poor prognosis group (*n* = 44)	Ρ
Male sex	Male	52	42	0.294
	Female	7	2	
Smoking status	No	45	33	0.882
	Yes	14	11	
Mechanism of injury	Low-energy blunt trauma	10	4	0.021
	High-energy blunt trauma	36	18	
	Crush injury	10	14	
	Other	3	8	
Open injury	No	43	18	0.001
	Yes	16	26	
Fracture	No	8	12	0.082
	Yes	51	32	
Blister	No	43	38	0.099
	Yes	16	6	
Dehydrating agent	No	14	19	0.036
	YES	45	25	
Time to fasciotomy	<6 h	8	2	0.252
	6 ~ 24 h	36	27	
	>24 h	15	15	
Arterial injury	No	48	4	<0.001
	Yes	11	40	
Debridement	0	10	2	0.005
	1 ~ 2	41	25	
	≥ 3	8	17	
VAC	No	32	22	0.67
	Yes	27	22	

**Table 2:** Bivariate Analyses of Measurement Data in Patients with ACS of lower leg

Item	Good prognosis group (*n* = 59)	Poor prognosis group (*n* = 44)	Ρ
Age(year)	43.00(28.50, 53.50)	36.00(27.75, 49.50)	0.270
Time to hospital (hour)	7.00(5.00, 12.00)	7.00(5.00, 14.75)	0.586
HB (g/L)	125.13 ± 23.60	106.31 ± 26.69	<0.001
PLT(10^9^/L)	205.21 ± 60.63	193.86 ± 79.06	0.411
WBC(10^9^/L)	13.15(10.78, 16.77)	16.75(12.31, 21.58)	0.008
PT (S)	12.50(11.95, 13.42)	13.35(11.80, 14.40)	0.077
INR	1.10(1.03, 1.19)	1.10(1.06, 1.24)	0.199
APTT (S)	28.50(26.95, 31.00)	27.70(25.68, 31.53)	0.289
FIB (g/L)	2.54(2.06, 3.10)	2.27(1.80, 2.95)	0.156
ALT (U/L)	29.00(22.00, 49.00)	34.00(24.50, 68.75)	0.162
AST (U/L)	36.00(27.00, 60.00)	50.50(28.00, 145.25)	0.079
ALB (g/L)	37.36(34.30, 42.31)	33.15(25.28, 37.13)	<0.001
GLU (mmol/L)	6.80(6.01, 8.36)	7.61(6.44, 9.54)	0.073
CK at presentation (U/L)	756.40(376.50, 1600.00)	1760.85(535.25, 6624.25)	0.015
CK at peak (U/L)	1509.00(675.00, 3555.15)	11636.00(5358.25, 21995.85)	<0.001
NA (mmol/L)	137.60 (135.35, 140.00)	137.60(135.00, 139.48)	0.912
K (mmol/L)	3.93 ± 0.49	3.99 ± 0.62	0.594
CA (mmol/L)	2.18(2.09, 2.24)	2.03(1.84, 2.15)	0.004
HOD (day)	30.00(24.50, 47.00)	40.00(25.50, 61.75)	0.090

**Table 3:** Logistic regression analysis results

	OR	OR 95% CI		P
		Lower Confidence Limit	Upper Confidence Limit	
Low-energy blunt trauma High-energy blunt trauma Crush injury Other Open injury	0.490 2.764 1.166 3.146	0.40 0.154 0.027 0.612	5.964 49.484 50.916 16.167	0.375 0.575 0.490 0.027 0.170
Arterial injury	66.172	10.536	415.611	<0.001
Dehydrating agent	1.189	0.229	6.180	0.837
Debridement 0				0.910
Debridement 1 ~ 2	0.578	0.038	8.819	0.693
Debridement ≥ 3	0.500	0.021	12.007	0.669
HB	0.934	0.891	0.979	0.005
WBC	1.335	0.978	1.317	0.095
ALB	1.082	0.909	1.287	0.375
CK at presentation	1.000	1.000	1.000	0.320
CK at peak	1.000	1.000	1.000	0.856
Constant	3.286			

**Table 4:** ROC curve analysis and cut-off value of HB

Item	AUC	Cut-off value	Sensitivity	Specificity	95%CI		p value
					Lower limit	Upper limit	
HB	0.702	102.45	0.477	0.847	0.601	0.804	< 0.0001

## Discussion

ACS of the lower leg is an emergency clinical condition that occurs as a result of trauma or other factors leading to reduced limb perfusion. If adequate perfusion cannot be promptly restored, a poor outcome occurs. Vaillancourt showed that ischemia from ACS can cause muscle necrosis before 3 h elapses post-trauma [23]. In this study, we found that arterial injury and lower HB concentration were the primary reasons leading to bad outcomes in 44 patients, which confirmed the importance of limb perfusion. The timely and effective restoration of tissue perfusion can effectively improve patient prognosis. From another perspective, lower hemoglobin levels also reflect the severity of the patient’s injury.

Previous articles have also reported other factors that lead to one or more types of poor outcomes. Mortensen identified open fractures as the only independent risk factor for muscle necrosis by retrospectively reviewing 357 patients with ACS in the lower leg [17].

Dafang Zhang showed that older age, a higher modified Charlson Comorbidity Index score, higher potassium (K) levels, lower hemoglobin (HB) levels, and higher lactate levels were associated with death, and diabetes mellitus, absence of a compartment pressure measurement, greater partial thromboplastin time (PTT), and higher albumin (ALB) levels were associated with limb amputation [7]. Hope analyzed the data from 151 ACS patients (including 90 patients with ACS in the lower leg) and reported that muscle necrosis occurred more frequently in patients without fractures than in those with fractures [9]. In a retrospective study of 40 patients who underwent fasciotomy for ACS of the lower leg, Heemskerk reported that the only significant predictive determinant of amputation or death was the age of the patient [3]. To the best of our knowledge, few studies have focused on the risk factors for multiple poor outcomes after ACS of the lower leg. After reviewing the related articles and considering that any adverse outcome can affect patient gait after recovery, we conducted this at our hospital and focused on multiple types of adverse outcomes, with the aim of offering a more practical reference for orthopedic surgeons. To avoid interference from confounding factors, 14 patients who died were excluded from the analysis because they often had multiple severe injuries that could have impacted the outcomes. The cause of death is usually multiple severe injuries rather than ACS. Additionally, we included the risk factors mentioned in the articles above to the extent possible in the statistical analysis in the present study to identify those with the greatest impact.

The occurrence of limb amputation in patients with ACS of the lower leg has previously been reported by several researchers. Reported rates of limb amputation range from 5 to 21% [5, 7, 24, 25]. The rates of limb amputation (14.6%) in our study support these prior findings. Hope reported that 20% of patients without a fracture had muscle necrosis, whereas 8% of patients with a fracture had muscle necrosis. Mortensen reported that 14.3% of patients had muscle necrosis. Our study revealed that 44 (43.7%) of the 103 patients had poor outcomes. In the context of the findings of previous studies, our findings are not unexpected because nerve damage can lead to poor outcomes other than muscle necrosis, which suggests that nerve damage caused by ACS of the lower leg should not be ignored.

Early fasciotomy is generally considered to be the gold standard treatment for preventing poor outcomes. However, delayed fasciotomy due to missed diagnosis of ACS occasionally occur. The cases of delayed fasciotomy are frequently mentioned in the literature from the last century as being associated with poor outcomes [26–28]. However, in recent years, several scholars have shown that the time from injury to fasciotomy does not significantly affect the prognosis. Dafang Zhang reported that the time to fasciotomy was not associated with death or limb amputation in patients with acute compartment syndrome [7]. Mortensen reported no difference in the duration from injury to fasciotomy between patients with muscle necrosis and patients without muscle necrosis [17]. The last two articles above had larger sample sizes and more comprehensive statistical analyses than earlier articles did; these findings have more value as a reference. Our research led us to the same conclusion. Mubarak suggested that the relationship between time since injury and compartment pressure remains controversial, and it is difficult to determine the exact time of the onset of symptoms in a patient unless compartment pressures are measured to substantiate the clinical impression [29]. Even though fasciotomy is performed, tissue perfusion may not be restored due to arterial injury or arterial embolism. This may explain why the time from injury to fasciotomy was not found to be associated with poor outcomes in our research. In addition, although there was not enough evidence to support our hypothesis, clinical observation revealed that blisters can still be considered a reliable mechanism by which pressure is released after trauma. Due to technical limitations, we were unable to observe a relationship between the time of occurrence of blisters and pressure fluctuations.

To date, there is no gold standard for the diagnosis of ACS, and usually, the determination of a diagnosis of ACS is based on surgeons’ clinical experience. Although 5Ps, pain out of proportion, pallor, paresthesia, paralysis, and pulselessness, have been defined as the clinical hallmarks of ACS, the clinical symptoms are not reliable because these signs may not occur in patients with ACS. Todd reported that none of the patients diagnosed with ACS had three or more clinical symptoms [30]. Although most orthopedic surgeons agree that the measurement of compartment pressure is a viable method, this approach is not always feasible. There is no uniform format for the instruments or methods used for measuring compartment pressure, and most hospitals do not have the technical equipment. In reviewing the patients’ medical records, we observed that some of them had undergone angiography of the lower limbs. In patients without arterial injury, the perfusion rate of the contrast agent was significantly slower than that in patients without ACS. Even during contrast agent perfusion, reflux occurs. This phenomenon suggested a lack of blood supply to the lower leg and the need for a fasciotomy. As a mature technique, angiography is carried out in many hospitals. The author believes that the findings of the above phenomena can provide an important reference suggesting the diagnosis of ACS. In addition to providing evidence that can support a diagnosis, angiography after fasciotomy can also reveal tissue perfusion in the lower leg.

The cause of, damage due to, and prognosis of ACS vary greatly among patients, and a practical classification of ACS is still lacking. The ICD-9 diagnosis codes merely classify ACS of the lower extremity as traumatic or nontraumatic, which is far from meeting the requirements of surgeons. ACS of the lower leg may be caused by soft tissue injury (crush injuries and open injury), fracture (sometimes accompanied by overly tight plaster fixation) or arterial injury. In this study, we did not find the fracture have any influence on the outcome, univariate analysis revealed statistically significant differences in the mechanism of injury (including crush injury), open injury and arterial injury between the two groups; however, multivariate logistic regression analysis revealed that arterial injury was the only independent risk factor for a poor prognosis. The risk of poor outcomes in patients with arterial injury was 66.172 times greater than that in patients without arterial injury. Therefore, we believe that the classification of ACS in the lower leg should be based on the presence or absence of arterial injury, as this classification can predict the prognosis and provide a reference for treatment.

Several limitations of this study should be noted. First, it is unavoidable that using data from a single center study affects the accuracy of the results, and a multicenter study is needed. Second, retrospective studies inevitably have inherent limitations.

with regard to data collection. Third, because of the limited number of patients observed, arterial injury was not further classified in this study, and amputation, neurological deficit and contracture were not analyzed separate outcomes. Solving this problem requires additional studies with larger sample sizes. Fourth, we did not have a unified standard for the diagnosis of ACS, all diagnoses are based on the clinical experience of different surgeons. We will look for a set of unified and effective standards in future studies. In addition, in the patients with arterial injury, we could not determine whether the sequelae were caused by ischemia or reperfusion injury.

This study emphasizes the importance of monitoring arterial injury and low HB concentration in ACS patients. Timely artery repair and suitable blood transfusion are crucial to prevent bad prognosis.

## Conclusion

ACS of the lower leg is a serious complication often associated with a poor prognosis. Patients with arterial injury or lower HB have a significantly increased risk of having poor outcomes. Poor outcomes were not found to be associated with the timing of fasciotomy in this study.

## Data Availability

No datasets were generated or analysed during the current study.

## Abbreviations

ACS: acute compartment syndrome
GG: the good prognosis group
PG: the poor prognosis group
HB: hemoglobin levels
PLT: platelet count
INR: international normalized ratio
WBC: white blood cell count
PT: prothrombin time
APTT: activated partial thromboplastin time
FIB: fibrinogen
ALT: alanine aminotransferase levels
AST: aspartate aminotransferase levels
ALB: albumin levels
GLU: glucose levels
CK: creatine kinase levels
Na: sodium levels
K: potassium levels
Ca: calcium levels
VAC: negative pressure wound vacuum assisted closure
